# Roles of Low Temperature Sputtered Indium Tin Oxide for Solar Photovoltaic Technology

**DOI:** 10.3390/ma14247758

**Published:** 2021-12-15

**Authors:** Susana Fernández, José Pablo González, Javier Grandal, Alejandro F. Braña, María Belén Gómez-Mancebo, José Javier Gandía

**Affiliations:** 1 Departamento de Energía, Centro de Investigaciones Energéticas, Medioambientales y Tecnológicas (CIEMAT), Avenida Complutense 40, 28040 Madrid, Spain; JosePablo.Gonzalez@ciemat.es (J.P.G.); jj.gandia@ciemat.es (J.J.G.); 2 Instituto de Sistemas Optoelectrónicos y Microtecnología (ISOM), Departamento de Electrónica Física, Universidad Politécnica de Madrid, Avenida Complutense 30, 28040 Madrid, Spain; javier.grandal@upm.es; 3 Grupo de Electrónica y Semiconductores, Universidad Autónoma de Madrid, Avenida Francisco Tomás y Valiente 7, 28049 Madrid, Spain; alejandro.brana@uam.es; 4 División de Química, Centro de Investigaciones Energéticas, Medioambientales y Tecnológicas (CIEMAT), Avenida Complutense 40, 28040 Madrid, Spain; mariabelen.gomez@ciemat.es

**Keywords:** magnetron sputtering, electron transport layers, cost-effective, indium-saving multicomponent oxides, photovoltaic applications

## Abstract

Different functionalities of materials based on indium tin oxide and fabricated at soft conditions were investigated with the goal of being used in a next generation of solar photovoltaic devices. These thin films were fabricated in a commercial UNIVEX 450B magnetron sputtering. The first studied functionality consisted of an effective n-type doped layer in an n-p heterojunction based on p-type crystalline silicon. At this point, the impact of the ITO film thickness (varied from 45 to 140 nm) and the substrate temperature (varied from room temperature to 250 °C) on the heterojunction parameters was evaluated separately. To avoid possible damages in the heterojunction interface, the applied ITO power was purposely set as low as 25 W; and to minimize the energy consumption, no heat treatment process was used. The second functionality consisted of indium-saving transparent conductive multicomponent materials for full spectrum applications. This was carried out by the doping of the ITO matrix with transition metals, as titanium and zinc. This action can reduce the production cost without sacrificing the optoelectronic film properties. The morphology, chemical, structural nature and optoelectronic properties were evaluated as function of the doping concentrations. The results revealed low manufactured and suitable films used successfully as conventional emitter, and near-infrared extended transparent conductive materials with superior performance that conventional ones, useful for full spectrum applications. Both can open interesting choices for cost-effective photovoltaic technologies.

## 1. Introduction

Transparent conductive oxides (TCOs) are attracted increasing interest as essential components for successful development of a wide range of optoelectronic devices and energy harvesting applications, such as touch screens, flat panels, solar photovoltaic cells, light-emitting diodes, sensors or low emissivity windows [1,2,3]. In several applications, low-cost TCO materials must be used because the device technology requires specific material properties. Examples of the mostly used TCOs are indium tin oxide (ITO), aluminum-doped zinc oxide (AZO), cadmium oxide (CdO) or tin oxide (SnO_2_) [4,5,6]. For all these materials, high performance in both visible-range transparency and conductivity, simultaneously, are highly desirable. The coexistence of both properties basically depends on its nature, its number and atomic arrangements of metal cations, its morphology and its intrinsic and/or introduced defects. Thanks to these properties, TCOs present several and different roles on the devices; as example, they are an essential and crucial component in photovoltaic (PV) technology [7,8,9,10]. This is due to the TCO properties exert a strong influence on the PV cell parameters such as the open-circuit device voltage V_OC_ or the short-circuit current J_SC_, contributing directly to the device performance. TCOs in PV can act as (i) transparent electrodes, (ii) structural templates and/or (iii) diffusion barriers. The most widely used TCO material remains being to be ITO despite its relative scarcity that increases the price of indium and its relative high consumption-energy manufacturing process [11,12]. So far, no TCO has succeeded improving its optoelectronic properties. 

Deposition techniques to fabricate ITO films are well developed, being magnetron sputtering one of the most desirable [11]. It provides a good adherent material with high performance to manufacture at high deposition rates at industrial scale and with the ability to control the film quality. In addition, this technique also permits thin film deposition at low temperature. All these features make it one of the most suitable to manufacture competitive TCO materials. Despite this, manufacturing high-quality ITO films at low temperature is still a challenge [13,14]. In this sense, intensive investigations have been carried out, showing high quality ITO layers deposited at room temperature (RT) by controlling parameters as oxygen flow [9], or by using soft sputtering process thanks to decrease the direct-current (DC) power values applied [15]. However, even with these achievements, the high cost of indium remains being a problem that motivates to investigate other substitute materials [16,17,18,19,20]. In this sense, the cost-reduction of conventional ITO electrodes by indium-saving is attracting considerable attention. By mixing or co-sputtering low-cost metal oxides within an ITO matrix, the indium content in indium-based TCO electrodes can be reduced, and therefore, the indium consumption. Examples of it can be TCO multi-components as In–Zn–Sn–O, In–Al–Zn–O, In–Al–Zn–Sn–O, Ga-Ti-In-O or even In–Ga–Zn–O that show very smooth surfaces and excellent optical properties, with transmittances higher than 80% in the visible range and low resistivities in the range of 10^−4^ Ω-cm [17,20]. In addition, titanium doping on In_2_O_3_ can improve the mobility reaching values higher than 100 V^−1^ s^−1^ cm^2^ that would help to the carrier transport, enhancing the device efficiency [16,19]. Moreover, the problem with the scarcity of indium is currently solved by the recycling. In this sense, many companies are offering programs to recycle this material [21], following the recommendations of the new environmental policy of circular economy [22]. However, if these environmental policies do not apply soon, an indium shortage may occur in the next few years. 

On the other hand, in most of the manufactured products that incorporate ITO in the production chain, post-deposition heat treatments are required. They are necessary to achieve high-quality ITO thin films with adequate electronic properties retaining high transparency [13]. In the PV sector, the next device generation demands cost-effective products by reducing energy and avoiding material losses in the manufacture process. Low-temperature fabrication processes compatible with thinner and/or lower-quality wafers are desired [23]. Under this premise and trying to adapt to the demand of the new technologies, this work shows the optimization of ITO based materials at low energy consumption regime to be used in different scenarios: (i) as an effective N-doped layer in a n-p heterojunction acting as a selective carrier layer. For this purpose, the ITO films were deposited in an oxygen-free environment at soft sputtering conditions and without any post-annealing treatment to reduce the energy manufacturing consumption and, therefore, to diminish the production cost; and (ii) as an indium-saving multicomponent TCO by the doping with transition metals, as titanium and zinc, to replace the conventional antireflective (AR) transparent electrodes. These kinds of materials can be very useful for both the mature and the next PV technologies. In this case, what is intended is to reduce the cost of ITO electrodes thanks to the doping. Finally, by controlling the amount of compounds ratio, the optoelectronic properties of these multicomponent can be easily adjusted as function of device requirements.

## 2. Materials and Methods

ITO thin films were deposited in a commercial UNIVEX 450B system from Leybold (Leybold GmbH, Cologne, Germany). This sputtering system is equipped with four magnetron sources placed in a confocal geometry with respect to the substrate holder, distanced from each other about 15 cm. This confocal configuration helps to ensure film homogeneity. Two of the four magnetrons are operated by Radio Frequency (RF) and the other ones, by direct current (DC). For ITO film deposition, 4-inch diameter SnO_2_:In_2_O_3_ ceramic target provided by Neyco, Vanves, France was placed on a direct current (DC) source. The nominal target composition was 10:90 wt% and its purity was 99.99%. Finally, the ceramic ZnO:Al_2_O_3_ (98:2 wt%) and TiO_2_ targets used for the doping, both from Neyco, Vanves, France, had a purity of 99.95% and both were operated by RF.

Depending on the ITO role studied, different substrates were used: (1) resistive Corning glass (Corning Inc., New York, USA) to evaluate its optical properties in the visible (VIS) and near-infrared (NIR) range, (2) polished resistive float zone (FZ) <100> silicon wafer (resistivity >10^4^ Ω-cm) (Topsil, Frederikssund, Dinamarc) to determine its AR capability and electrical conductivity, and (3) double double-polished FZ p-type silicon <100> wafers (resistivity ~1000–5000 Ω-cm) (Topsil, Frederikssund, Dinamarc) as absorber in the n-p heterojunction. 

The sputtering process of the ITO-based thin films was performed with a base pressure of around 10^−5^ Pa in an oxygen-free environment. 9N5 Argon (Ar) was the inert gas used, and its flux was controlled by an MKS mass flow controller (MKS Instruments, Andover, MA, United States). During the sputtering process, the substrate was rotated at 20 r.p.m. The gas flow rate and the working pressure were set to 5 sccm and 0.17 Pa, respectively. The DC power (DCP) values were varied from 25 to 75 W, and the substrate temperature, from RT to 250 °C. 

Finally, the co-sputtered samples were simultaneously deposited at a DCP varied from 0 to 300 W for ITO target, and at a constant value of 250 W for the RF power (RFP) applied to AZO target; while the RFP values of the TiO_2_ target varied between 25 and 50 W. The Zn-doped ITO films were deposited at RT; meanwhile, the Ti-doped ones, at 450 °C. 

The structural composition of thin films was determined by X-Ray diffraction (XRD). The X-ray spectra were obtained with a PANalytical X’Pert Pro diffraction system with a vertical Thetha wide-angle goniometer (Malvern Panalytical Ltd., Malvern, UK), operating in grazing incidence (GI) configuration. At this configuration, a Goebel-type parallel beam mirror on the incident beam side and a linear X’Celerator detector (Malvern Panalytical Ltd., Malvern, UK) in receiving slit mode attached to a parallel plate collimator on the diffracted beam side were used. The radiation used was CuKα (45 kV–40 mA), at a fixed incident angle of ω = 2.5°, in parallel beam geometry in an angular range of 20° < 2θ < 80°. Phase identification was obtained by comparison with The Inorganic Crystal Structure Database (ICSD). To determine the elemental composition of ITO layers, wavelength dispersive X-ray fluorescence analysis (WD-XRF) was carried out with a PANalytical AXIOS automated XRF spectrometer (Malvern Panalytical Ltd., Malvern, UK). The samples were analysed by means of a semiquantitative (OMNIAN) method developed by PANalytical. Film morphology was determined by using a Digital Instruments Nanoscope IIIa Atomic Force Microscope (AFM) (CSI instruments, Les ULIS, Paris, France) in tapping mode with Bruker NCHV probes. The scans were processed using Nanotec software WSxM [24]. Hall mobility and carrier concentration were obtained using the Van der Pauw method under a 1.2 T magnetic field at RT. Optical transmittance (T) at the 300 to 2500 nm wavelength range was measured at RT and normal incidence with a UV/Visible/NIR Perkin-Elmer Lambda 1050 spectrophotometer (Waltham, MA, USA). The optical band gap (E_GAP_) values for direct band gap semiconductors were estimated by Tauc’s Equation (1)

(αhν)^2^ = A (hν − E_GAP_) (1)

where α is the absorption coefficient, hν is the photon energy and A is a parameter proportional to the probability of electron transition between empty and occupied states [25]. The E_GAP_ can be determined by extrapolation of the linear part of (αhν)^2^. Additionally, the AR capability was also determined from the hemispherical reflectance spectra measured with the above-mentioned spectrophotometer and using the 6 mm integrated sphere accessory (Waltham, MA, USA). 

In this work, ITO-based n-p heterojunction photocells were fabricated onto the polished FZ p-type silicon substrates. In this design, ITO layer plays the main role of the n-type emitter. Prior to its fabrication, the silicon wafer was dipped in HF (Mervilab, Madrid, Spain) to remove the native oxide just before loading it into the sputtering chamber. Lastly, a metallic grid of Ti (55 nm)/Ag (1 µm) (UMICORE, Brussels, Belgium) was evaporated as front contact, and 0.9 µm-thick aluminum (UMICORE, Brussels, Belgium), as rear contact, that was previously annealed at 350 °C for 2 h in an Argon environment to achieve an ohmic behavior. The current-voltage (JV) characteristics of the devices were measured under illumination calibrated at AM1.5G conditions and 100 mW/cm^2^, using a class A solar simulator (Steuernagel SC575) (K.H. Steuernagel Lichttechnik GmbH, Morfelden-Walldorf, Germany). 

## 3. Results

In the following subsections, we describe the main results corresponding to each role of ITO.

### 3.1. Role of ITO as Effective N-Layer in a n-p Silicon Heterojunction Photocell

In this subsection, we investigate how ITO sputtering process itself influences on the main electrical parameters of a n-p heterojunction photocell. In particular, the effect of the ITO thickness, ranged from 45 to 140 nm, and the film nature are both tested. These ITO thin films were deposited at RT, 25 W of DCP, and 0.17 Pa of Ar working pressure, without post-annealing treatment. 

Lastly, the influence of ITO temperature deposition on the heterojunction properties is also checked as function of the film nature. In this case, the substrate temperature was ranged from RT and 250 °C; the rest of parameters was set to 25 W and 0.17 Pa, while the layer thickness was close to 45 nm. 

#### 3.1.1. Effect of ITO Thickness and Film Nature

**Figure 1:**
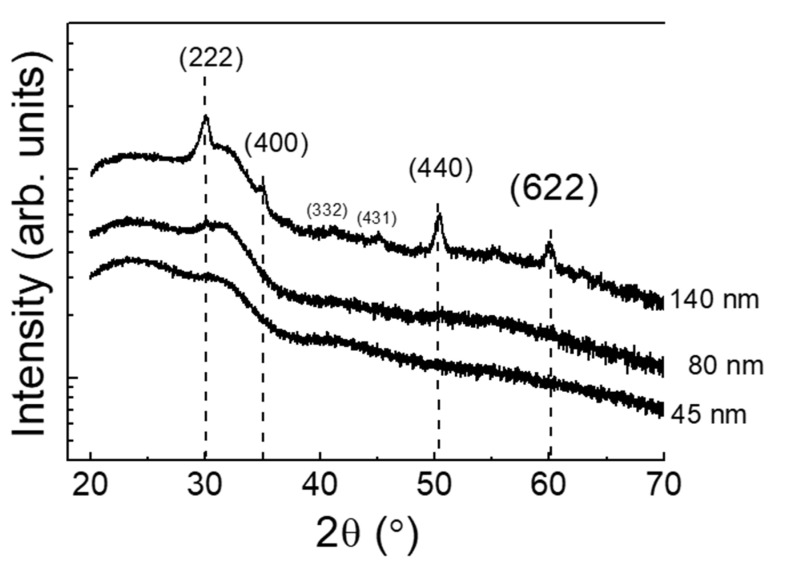
GI-XRD scans of ITO thin films as function of the thickness, deposited at soft sputtering conditions.

GI-XRD scans of as-deposited ITO thin films as function of thickness are depicted in Figure 1. A transition from amorphous to polycrystalline (pc) nature was observed at films thicker than 80 nm. A broad shoulder, that corresponds to the amorphous glass, hides a very weak peak around 30° that is noticeable for samples with ITO films thicker than 80 nm. The 140 nm-thick film spectrum showed four main peaks related to the (222), (400), (440) and (622) planes, respectively. The (222) plane, corresponded to the characteristic structure of cubic bixbyite In_2_O_3_ phase (Card no. 65–3170), presented the maximum intensity indicating the preferred orientation of this film. This means that ITO films deposited at RT on glass need to be thicker than others deposited at higher temperature or on other crystalline substrate as silicon, as other authors reported previously [26]. The appearance of (222) cubic reflection in the thickest studied film is attributed to the greater mobility that the atoms possess while growing along the parallel direction to the substrate surface when thickness increases, and hence, an improvement of the crystallisation process is reached [27]. No characteristic peaks of tin (Sn), SnO or SnO_2_ appeared in the scans, meaning that the hosting of Sn in the substitutional position of the In_2_O_3_ cubic lattice occurred successfully [13]. 

The elemental composition (in percentage) was semi-quantitatively estimated from WD-XRF measurements. No significant dependence of the composition was found with the thickness and film nature. The percentage of In_2_O_3_ and SnO_2_ remained almost constant to 87.8 ± 1.5 and 12.2 ± 1.0, respectively, slightly different than the nominal composition of the ceramic target (90:10 wt%).

Table 1 shows the electrical parameters (carrier density n, mobility µ, sheet resistance R_sh_ and resistivity ρ), obtained from Hall measurements, as function of the ITO thickness.

The film nature and the (222)/(400) peak intensity ratio obtained from XRD patterns were also included in Table 1. A drastic drop in the carrier concentration and a sharply increased in the mobility were observed in the thickest sample with pc nature. The trend in both electrical parameters could be due to the different film nature. In general, in ITO films, both Sn dopants and ionized oxygen vacancy donors provide the charge carriers for conduction. However, depending on its nature, it is well known that Sn doping did not contribute generating carriers when the film is amorphous [28]; in the case of the pc films, the Sn atoms diffused from interstitial to In cation sites, producing a realignment of In-O bonds and thus, generating a locally ordered structure. In this case, the low resistivity is attributed to an enhancement in the mobility, closely related to the crystalline improvement observed in XRD scans, and to the effect of the lattice modifications due to the doping that occurs when thickness is increased [29,30]. Hence, the mechanism of providing carriers is different, and hence, no relationship can be established at the transition point. 

Table 2 collects the normalized average transmittance values in the visible (400–800 nm) T_VIS_, and in the NIR (800–2500 nm) wavelength region T_NIR_, as function of the thickness. The band gap energy E_GAP_ and the film nature are also included.

A clear opaqueness with thickness increasing was observed by the reduction of T_VIS_ value just up to the transition point. Above it, the value remained almost constant. This could be attributed to the different nature of the layer and the ordering/realignment of the oxygen that may take place in the pc film. Regarding the change of T_NIR_, a clear sharp drop was observed as a function of the layer thickness. In the case of the sample at the transition point, that decrease can be attributed to the observed increase of the carrier concentration (see Table 1), and hence, an enhancement of the free-carrier absorption occurred. On the other hand, the decrease of T_NIR_ in the pc film could be attributed to the combined effect of the change in the nature of the film and its superior electrical resistivity. 

**Figure 2:**
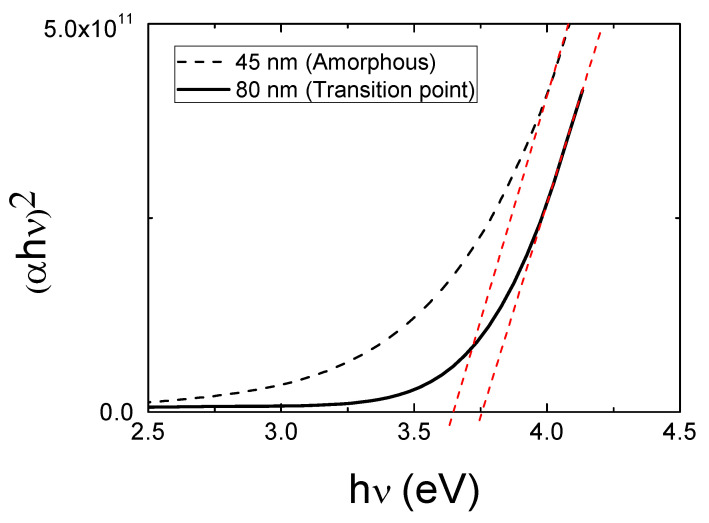
$(\alpha h\nu {)}^{2}$ versus photon energy (hν) used to calculate the band gap energy values of the thin films at the transition from amorphous to pc nature.

Figure 2 plots $(\alpha h\nu {)}^{2}$ vs photon energy (hν) used to calculate the band gap energy E_GAP_ for the 45 and 80 nm thick films. The extrapolation of the straight-line portion of the plot of $(\alpha h\nu {)}^{2}$ to zero absorption (red lines in the graph) gives the direct band gap of the film. As it can be appreciated, a clear blueshift was observed as film thickness increases. In agreement with other authors, we found a broader E_GAP_ as thickness increased [31]. This is related to the Burnstein-Moss phenomenon [32] and hence, closely related to the high carrier concentration measured in the sample at the transition point. In the case of the pc film, the broaden of the E_GAP_ could be attributed to a change in its crystalline nature [33]. 

**Figure 3:**
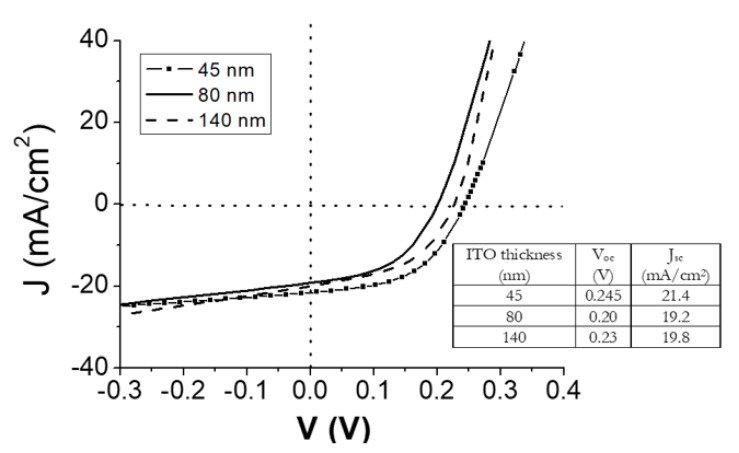
J-V characteristics measured under illumination of the photocells fabricated with ITO thin films with different thickness. In the inset, the table with the electrical parameters V_OC_ and J_SC_.

Figure 3 depicts the J-V characteristics measured under the illumination of the photocells fabricated with ITO films of different thicknesses. It should be highlighted that no passivation layer was used in the devices to directly evaluate the capability of ITO as carrier transport layer. Due to that, the Voc values were much lower than the commonly obtained in the silicon-based technology (close to 700 mV). A strong influence on Voc was observed varying the ITO thickness in the range of 45 to 140 nm, as it’s shown in Figure 3. The highest value of V_OC_ was achieved incorporating 45 nm-thick ITO amorphous layer into the photocell. This can be related to the film mobility, and it should be indicative of the effective transport of photogenerated electrons. Therefore, the difference observed in the V_OC_ of devices fabricated with 45 nm-thick (amorphous layer) and 140 nm-thick films (pc layer) (see the V_OC_ values in the inset of Figure 3) can be related to (i) the nature of the ITO film, (ii) the effect of grain boundary scattering in the pc-film and (iii) the band alignment modified with the carrier concentration [34,35]. Even the amorphous film presented lower mobility than the pc one (20.2 cm^2^/Vs vs 25.5 cm^2^/Vs), this effect could be compensated by the non-existed boundary grains, and hence, scattering at them. Therefore, the electrons could flow easily from the active layer. Regarding the Jsc, no systematic trend was showed with ITO thickness, being slightly higher in the case of using a 45 nm-thick ITO layer.

As summary, the photocell with better performance was achieved using a 45 nm-thick ITO thin film deposited at RT, a soft DCP of 25 W, and a working pressure of 0.17 Pa. Under these conditions, values of V_OC_ and J_SC_ of 0.245 V and 21.4 mA/cm^2^, respectively, were obtained. This ITO film presented an amorphous nature that clearly favored the electrical performance of the heterojunction.

#### 3.1.2. Effect of Substrate Temperature

**Figure 4:**
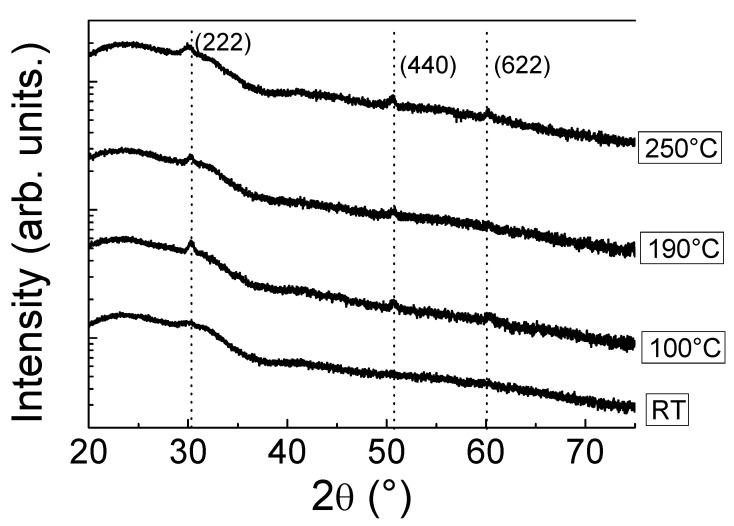
GI-XRD scans of 45 nm-thick ITO thin films as function of the substrate temperature.

Taking as starting point the previous result showed in the Section 3.1.1, we study the effect of increasing the ITO substrate temperature on the behavior of the heterojunction n-ITO/p-Si. Figure 4 depicts the GI-XRD scans of the 45 nm-thick ITO films deposited at 25 W, 0.17 Pa and different substrate temperatures, ranged from RT to 250 °C. As can be seen in the spectra, a weak pc structure was obtained as the substrate temperature increases, observable at 100 °C and above. Three weak diffraction peaks can be appreciated, related to the (222), (440) and (622) planes. The obtained diffraction peaks were in accordance with the standard JCPDS data (Card no. 65-3170). The low peak intensity was attributed to the low dimensionality of the films (~45 nm). In all cases, the (222) diffraction peak around 30° was hidden into a broad shoulder corresponding to the amorphous glass signal. Therefore, any change in its intensity is clearly observed when the substrate temperature was increased. These results indicate that an increasing in the substrate temperature helps to improve the adatom mobility on the substrate surface enhancing film crystallinity [36]. No characteristic diffraction peaks of Sn, SnO or SnO_2_ were noticeable in the scans, meaning the suitable hosting of Sn in substitutional position of the In_2_O_3_ cubic lattice.

**Figure 5:**
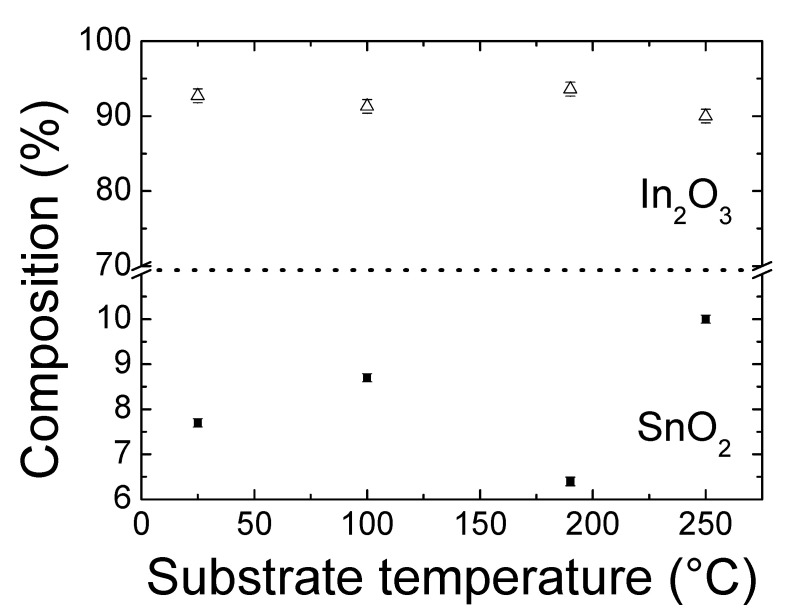
Compositional percentage of the In_2_O_3_ and SnO_2_ binary compounds estimated from WD-XRF measurements.

Figure 5 shows the chemical composition (in percentage) of the binary compounds estimated from WD-XRF measurements as function of the substrate temperature. No remarkable compositional changes were observed when the substrate temperature was increased, except for sample deposited at 250 °C. This layer showed a slight increase, and a corresponding decrease, in SnO_2_ and In_2_O_3_ concentrations, respectively. Despite these small fluctuations, the values were close to the nominal ones.

**Figure 6:**
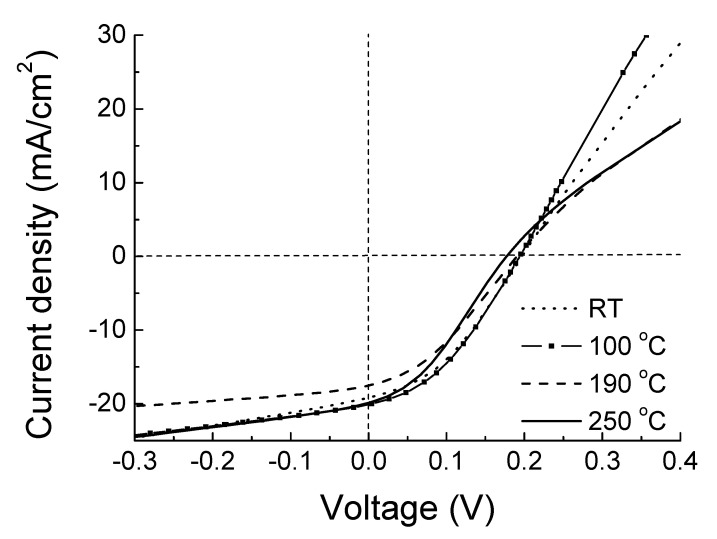
J-V characteristics measured under illumination of the photocells fabricated with ITO thin films fabricated at different substrate temperature.

Figure 6 shows the J-V characteristics under the illumination of the photocells fabricated with ITO films deposited at different substrate temperature. A rectifying behavior was obtained, regardless of the ITO thin film used. As happened in the samples of the previous subsection, no passivation layer was used in the photocell design. Table 3 summarizes the electrical parameters of the ITO layers, the film nature and the value of the open-circuit voltage V_OC_ (Figure 6), while Table 4 showed the optical parameters of ITO, the film nature and the short-circuit current J_SC_ value extracted from Figure 6. The error estimated for the measured electrical parameters V_OC_ and J_SC_ from Table 3 and Table 4 was close to 3%. 

The minimum of carrier concentration, 5.8 × 10^20^ cm^−3^, is observed when the ITO film was deposited at 100 °C to gradually increase again at higher temperatures. This may be attributed to an improvement of the Sn atoms diffusion into In cation sites as substrate temperature increased. In the pc films, Sn acts as a donor, which would lead to an increase in carrier concentration, as it was observed. On the other hand, at 250 °C of substrate temperature, it is possible that a slight excess of Sn doping, observed from WD-XRF measurements (see Figure 5), caused an increasing in the concentration of the electron traps that might act as carrier traps rather than electron donors, giving rise to a detrimental-to-the-mobility scattering process. With respect to the photocells, the highest V_OC_ of 0.197 V observed is obtained using an ITO thin film deposited at RT; a film whose nature was amorphous. This is related to the highest mobility presented in this amorphous film, corroborating its effectiveness as electron transport. 

**Figure 7:**
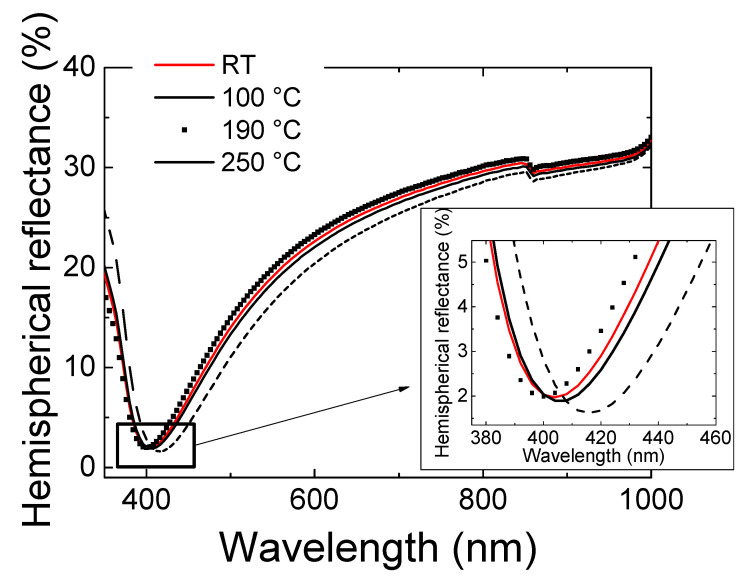
Hemispherical reflectance spectra of the photocells fabricated with ITO thin films deposited at different substrate temperature. In the inset, the zone in which the spectrum reaches a minimum.

On the other hand, the optical losses can influence on the Jsc parameter, extracted from Figure 6. These optical losses are due to the number of photons (i) reflected on the surface and (ii) absorbed by the ITO film through the free carriers. In both cases, those photons would not reach the silicon absorber. In this sense, Table 4 shows the lowest J_SC_ value of 17.6 mA/cm^2^, achieved in the photocell with a pc ITO film fabricated at 190 °C. This film presented the highest average hemispherical (total) reflectance value at the visible wavelength range of 23.9% (more reflection on its surface) and a highest value of the reflectance minimum at the wavelength of 400 nm, attributed to the free carriers’ absorption (see the inset in Figure 7). Both facts indicate that this film has the greatest optical losses. In any case, the minimal differences observed in J_SC_ values were mainly attributed to the small variations in ITO thickness, showed by the slight hemispherical reflectance spectra shift in Figure 7. 

These results showed that the increase of the substrate temperature did not enhance the electrical performance of the photocell, despite improving the optoelectronic properties of the ITO thin film. This can be explained because the structural enhancement, due to the rise of the temperature, led to the increase of both the carrier traps and the scattering process, resulting detrimental for the electrical performance of the n-ITO/p-Si heterojunction. In order to avoid this, an amorphous film fabricated at RT was preferred.

Finally, the feasibility of ITO film as an effective n-layer in a n-p heterojunction was demonstrated, overall, when ITO was fabricated at RT and soft sputtering conditions such as 25 W of DCP and 0.17 Pa of working pressure with thicknesses as thin as 45 nm. 

### 3.2. Indium-Saving Multicomponent Materials to Replace Conventional TCOs in Different Photovoltaic Technologies

One of the main challenges in the fabrication of SHJ solar cells is the achievement of low-cost and high-quality TCOs to (i) extract efficiently the charge carriers, (ii) act as AR coating by decreasing the light reflection on device surface, allowing a higher fraction of light may reach the silicon and (iii) reduce the production cost. In this sense, low-temperature manufacturing processes are being increasingly desired [37]. 

On the other hand, the next-generation photovoltaic technologies, such as organic solar cells (OSCs), require the anode layer, the organic active layer and cathode layer consisting of low-cost materials. Thereby, the replacement of conventional ITO by multicomponent materials based on annealed sputtered low-cost metal oxides embedded into an ITO matrix is gaining attention in that area [16]. 

In this subsection, seeking for TCO materials that obey the above-described conditions, we study the effect of doping the In_2_O_3_ matrix with transition metals such as Zn and Ti, respectively, to determine its suitability as possible substitutes for applications in different photovoltaic technologies. 

#### 3.2.1. Zn as Dopant in ITO Matrix

The multicomponents were deposited at RT and 0.17 Pa of working pressure on both Corning glass and polished resistive float zone <100> silicon wafer (resistivity > 10^4^ Ω-cm) to determine the optical and electrical properties, respectively. The nominal thickness of the In_2_O_3_-matrix-based films co-sputtered with the ceramic ZnO:Al_2_O_3_ (98:2 wt%) (AZO) target of 4-inch-diameter was set to 80 nm because of the SHJ technology specifications [38]. The DCP applied to ITO target varied from 25 to 300 W, while maintaining at 250 W the RF power applied to AZO one. The main objective is to achieve an indium-saving multicomponent material with suitable tunable optoelectronic properties and AR capability to replace the conventional TCOs for SHJ technology [39,40]. 

Table 5 collects the binary compounds concentrations of In_2_O_3_, ZnO, Al_2_O_3_ and SnO_2_ estimated from WD-XRF measurements, as function of the used sputtering target power. In order to validate the suitability of the multicomponent materials fabricated in this subsection, the data of the optimized TCOs used in SHJ technology are also collected as reference films, for comparison: i.e., ITO deposited at the soft conditions of 25 W and RT (#1) [15]; ITO deposited at 75 W and 190 °C (#2) [15] and AZO deposited at 250 W and 190 °C (#3) [41,42]. The film nature, determined by XRD (not shown here), is also included [15,42].

Data reveal the gradual substitution of In_2_O_3_ by ZnO as ITO DCP was reduced, from 7.0 wt% to 19 wt% at the lowest DCP value of 25 W. At the same time, as ITO DCP was increased, SnO_2_ was progressively displacing Al_2_O_3_, until it disappeared, fact that happened at the highest ITO DCP used value of 300 W. Most of them presented an amorphous nature in agreement with the phase diagram presented by other authors, except the sample deposited at 25 W ITO DCP and 250 W AZO RFP [43]. Regarding the optimized TCOs considered as reference, in all cases, the composition was very close to the nominal value of the targets. At the same time, the reference samples deposited at 190 °C showed a polycrystalline nature. This fact might be attributed to two reasons (i) the high kinetic energy of the atoms impacting due to the power values used, and (ii) the high surface diffusion due to the substrate temperature. Both, the power and the substrate temperature were high enough to achieve a crystalline structure. On the other hand, as the sputtering conditions of reference #1 are much softer, a crystalline phase was not obtained.

Finally, to evaluate the performance of layers described in Table 5, the Figure of Merit (FOM) was calculated by using Equation (2)

(2) $$\varphi_{TC}=\frac{T_{r}^{10}}{R_{sh}}$$

where $\;T_{r}$ is the transmittance at 550 nm and $R_{sh}$, the sheet resistance. The calculated FOM values are collected in Table 6, together with the AR capability determined from the average hemispherical reflectance at the visible wavelength range of 400–800 nm, and the percentage of saved indium, compared to that of the reference material (samples #1, #2 and #3). The FOM must be as high as possible, indicative of a superior layer quality, meanwhile the AR capability must be as low as possible, indicative of reduced optical losses.

Data from Table 6 reveals that the highest FOM of 2.23 × 10^−3^ Ω^−1^ was achieved by the multicomponent film deposited at RT, 0.17 Pa and 75 W of ITO DCP and 250 W of AZO RFP, respectively. This sample, with an indium-saving percentage of 7.5%, presented an amorphous nature and the lowest value of AR capability ~8.9 % that means that the optical losses due to the light reflection on the surface are minimized. In addition, its performance was superior to the optimized reference samples #1, #2 and #3. 

On the other hand, the multicomponent with the highest amount of indium-saving of 28%, deposited at 25 W of ITO DCP and 250 W of AZO, exhibited a relatively low AR capability of 11.5%, close to the values shown by the reference samples #1, #2 and #3, but, unfortunately, it had a very poor FOM, 0.35 × 10^−3^ Ω^−1^. This is attributed to its electrical detriment, i.e., a value of sheet resistance as high as 500 Ω/sqr and a very low mobility of 8.5 cm^2^/Vs as compared to the best sample, 90 Ω/sqr and 20.2 cm^2^/Vs. 

Despite the sample deposited at 150 W of ITO DCP and 250 W of AZO RFP showed a FOM close to the optimal one, the amount of indium-saved was very small, just 3.2%. Lastly, the reference sample #3, that had the highest In-saving of 100%, AZO (not based on In_2_O_3_-matrix), presented a slight worsening in the AR capability ~13%, and its FOM, 1.84 × 10^−3^ Ω^−1^, did not exceed the value of the sample considered as optimal. 

The topography images (not shown) [15,41,42] showed different surfaces depending on the material and its film nature. In the case of the reference samples an almost protrusion-free surface was observed for reference #1 (amorphous ITO deposited at 25 W and RT), a surface with many protrusions of different sizes and densities, for the reference #2 (pc ITO deposited at 75 W, 190 °C); and granular morphology without protrusions, for the reference #3 (pc AZO). On the other hand, the surfaces of multicomponent materials showed a protrusion-free homogenous granular morphology, with root-mean-square (RMS) values that decreased from 1.4 nm (pc) to 0.5 nm (amorphous) when ITO DCP increased. 

In summary, we achieved a multicomponent film with an indium-saving percentage of 7.5%, deposited at RT, 0.17 Pa and 75 W of ITO DCP and 250 W of AZO RFP, that showed superior optoelectronic properties that conventional TCOs: better AR capability, better FOM and a smooth surface free of protrusions that favors to be used as a transparent electrode. Hence, this material is capable of being a substitute for the conventional TCO materials, which would mean a cost-reduction of ITO electrodes for the SHJ technology.

#### 3.2.2. Ti as Dopant in ITO Matrix

Thin films based on the In_2_O_3_-matrix with a thickness of 80 nm were co-sputtered on Corning glass using a 4-inch diameter ceramic TiO_2_ target (purity of 99.99%) and the ITO target described previously at a substrate temperature of 450 °C and a working pressure of 0.45 Pa. According to the literature, high temperature deposition and/or annealing processes are often preferred to enhance the electrical performance of this kind of materials [44,45]. In this work, the DCP applied to ITO target was set to 50 W, while varying from 25 W to 50 W the RPF applied to TiO_2_ one. Such soft sputtering conditions were used to avoid a possible degradation of the subsequent device. The main goal is to find a material with a superior balance between the electrical and optical properties and with a high NIR transmission to replace conventional ITO in many applications that provides a better use of the full solar spectrum. 

Table 7 shows the binary compounds concentrations of In_2_O_3_, TiO_2_ and SnO_2_, estimated from WD-XRF measurements, as a function of the sputtering target power values. 

**Figure 8:**
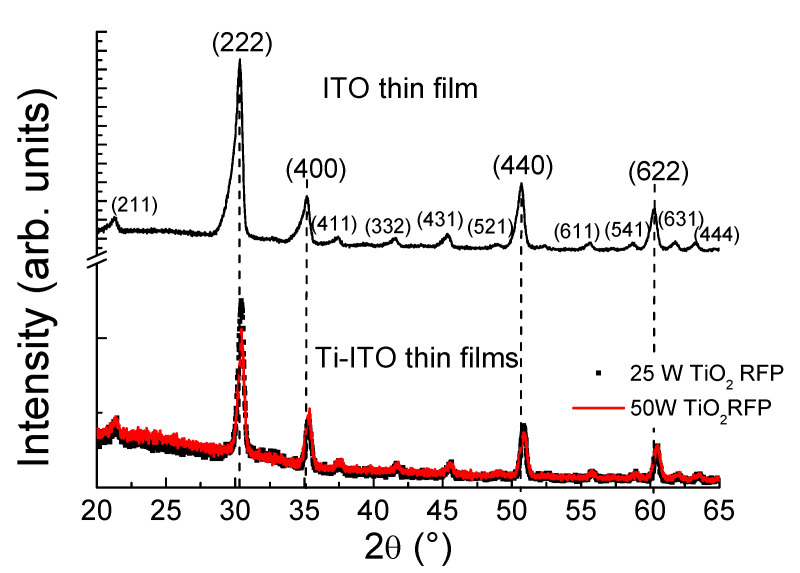
XRD pattern of the films in study.

The structural parameters were analyzed by XRD. The patterns depicted in Figure 8 revealed a polycrystalline nature with a bixbyite structure, typical of the ITO matrix, with a predominant (222) peak, regardless the sputtering conditions used. Other diffraction peaks corresponding to the (400), (440) and (622) planes also appeared with moderate intensity. Less intense peaks such as (444), (611), (411), (431) and (521) were observed too, supporting the pc nature of the films. In Table 7, the (222)/(400) diffraction peak intensity ratio was included, revealing that it decreased as TiO_2_ RFP value increased. This is indicative of a slight loss of crystal quality as the Ti^4+^ ion dopant gradually placed on In^3+^ sites. At the same time, the patterns of Ti-doped ITO thin films were displaced to higher angles when the dopant is introduced into lattice. This is attributed to the decrease of lattice spacing due to the shorter Ti^4+^ ionic radius compared to the In^3+^ one, 0.602 Å and 0.92 Å, respectively. No diffraction peaks of Sn or other impurity phases were detected in the prepared samples.

The evaluation of the performance of the indium-saving multicomponent materials was carried out analysing the FOM and the transmittance at visible wavelength region (400–800 nm), T_VIS_, and at NIR region (780 nm to 2500 nm), T_NIR_. Table 8 collects all these data together with the amount of indium-saving and the resistivity values of the films.

Data reveal a superior performance for the multicomponent with the highest doping level, with an indium-saving percentage of 0.5%, deposited at 50 W of ITO DCP and 50 W of TiO_2_ RFP, in comparison with the non-doped ITO thin film. Despite the doping level was quite low, attributed to the soft sputtering conditions used, the optical and electrical improvements achieved were quite noticeable: (i) a FOM as high as 8.47 × 10^−3^ Ω^−1^, (ii) an enhancement of both visible light transmission, close to 90%, and (iii) high NIR transmission of 83.5%. 

**Figure 9:**
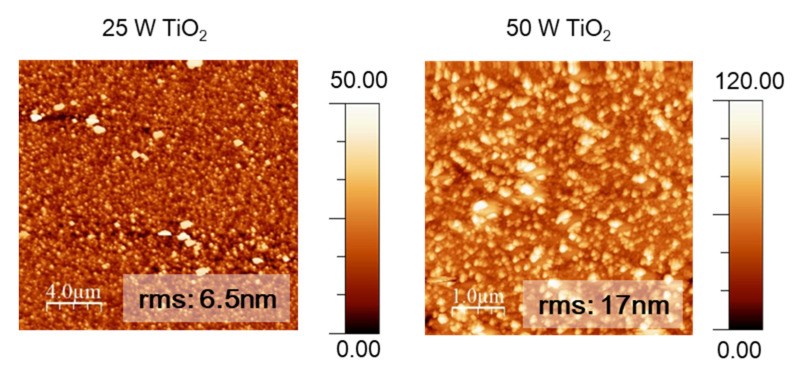
2D surface topography images of the multicomponent based on Ti-doped ITO matrix.

Figure 9 shows the 2D AFM micrographs of the Ti-doped ITO matrix. A rougher surface with an RMS value of 17 nm was obtained by the sample with the highest Ti concentration, indicative of a change in the morphology when the dopant was introduced into the lattice. 

As conclusion, we have demonstrated that the Ti doping can improve the optoelectronic properties of the ITO matrix, even using concentrations as low as 0.5 wt%. This co-sputtered film was deposited at 450 °C, 0.45 Pa and 50 W of ITO DCP and 50 W of TiO_2_ RFP. It showed values of FOM as high as 8.47 × 10^−3^ Ω^−1^ and a high NIR transmittance of 83.5% that would permit to provide a wider use of the solar spectrum. These results validate the Ti doping of ITO matrix in order to obtain a suitable material to replace conventional ITO in many applications and to provide a better use of the full solar spectrum.

## 4. Conclusions

In this work, several functionalities of ITO matrix were demonstrated: (i) as an effective N-doped layer in a P-type silicon-based heterojunction to replace conventional emitters, and (ii) as a suitable matrix to achieve an indium-saving multicomponent transparent electrode by the doping with transition metals to replace conventional TCOs. 

In the first case, the results showed an important influence of the sputtering conditions on photocell performance. The main limiting parameter was the nature of the ITO layer, determined by the deposition temperature and the power used. In this sense, the best device performance was achieved by using an amorphous ITO film with thickness as thin as 45 nm, deposited at RT, without any other heat treatment, 25 W of RFP and 0.17 Pa of working pressure. These results can permit to decrease the manufacturing cost by diminish the amount of material and reduce the energy consumption. 

On the other hand, the multicomponent materials based on the doping of the ITO matrix with Ti and Zn, respectively, were evaluated as suitable In-saving material. 

The results revealed that the multicomponents based on Zn-doping showed superior optoelectronic properties and good AR capability than conventional TCOs when its nature was amorphous, and were deposited at RT, 0.17 Pa and 75 W of ITO DCP and 250 W of AZO RFP. Under these conditions, the amount of indium-saving was around 7.5%. In the case of the multicomponent based on Ti doping, the data indicate the goodness of Ti dopant to achieve thin films with superior electrical properties and an enhanced VIS and NIR transmittance than the conventional TCOs used in the PV technologies. Incorporating Ti concentrations as low as 0.5 wt%, FOM as high as 8.47 × 10^−3^ Ω^−1^ with high NIR transmittances of 83.5% were reached at 450 °C, 0.45 Pa and 50 W of ITO DCP and 50 W of TiO_2_ RFP. Such both achievements permit opening the possibility to a noticeably improvement in the transparent electrode field and, therefore, to further enhance the different technologies.

## Figures and Tables

**Table 1:** Electrical parameters of the ITO films deposited at RT, 25 W and 0.17 Pa on resistive FZ silicon wafers. The nature of the film is also included.

ITO Thickness (nm)	Film Nature	I_(222)_/I_(400)_	n (10^20^ cm^−3^)	μ (cm^2^/V·s)	R_sh_ (Ω/sqr)	ρ (Ω·cm)
45	Amorphous	-	6.20	20.2	260	1.2 × 10^−3^
80	Transition amorphous to polycrystalline	-	7.20	16.0	85	6.8 × 10^−4^
140	Polycrystalline	2.17	4.20	25.5	55	7.7 × 10^−4^

**Table 2:** Optical parameters of the ITO films deposited at RT, 25 W and 0.17 Pa glass. The nature of the film is also included.

ITO Thickness (nm)	T_VIS_ (%)	T_NIR_ (%)	E_GAP_ (eV)	Film Nature
45	85.0	89.8	3.64	Amorphous
80	78.4	79.0	3.75	Transition amorphous to polycrystalline
140	79.6	69.8	3.98	Polycrystalline

**Table 3:** Main electrical parameters of the ITO films deposited at 25 W and 0.17 Pa as function of the substrate temperature. The nature of the films and the V_OC_ of the photocells are also included.

T_substrate_ (°C)	Film Nature	n × 10^20^ (cm^−3^)	μ (cm^2^/V·s)	R_sh_ (Ω/sqr)	ρ (Ω·cm)	V_OC_ (V)
RT	Amorphous	6.2	20.2	260	1.2 × 10^−3^	0.197
100	Transition amorphous to polycrystalline	5.8	15.0	200	9.0 × 10^−4^	0.194
190	Polycrystalline	6.0	18.6	180	8.1 × 10^−4^	0.190
250	Polycrystalline	5.9	16.0	170	7.6 × 10^−4^	0.177

**Table 4:** Main optical parameters of the ITO films deposited at 25 W and 0.17 Pa as function of the substrate temperature. The nature of the films and the J_SC_ of the photocells are also included.

T_substrate_ (°C)	Film Nature	T_VIS_ (%)	R_Hem_ (%)	J_SC_ (mA/cm^2^)
RT	Amorphous	85.0	23.3	19.2
100	Transition amorphous to polycrystalline	86.0	22.9	20.1
190	Polycrystalline	84.5	23.9	17.6
250	Polycrystalline	87.5	21.8	19.8

**Table 5:** Compound’s concentrations estimated by WD-XRF. The film nature is included.

ITO DC Power (W)	AZO RF Power (W)	Film Nature	In_2_O_3_ (wt%)	ZnO (wt%)	Al_2_O_3_ (wt%)	SnO_2_ (wt%)
25	250	Polycrystalline	67	19	11	3.5
75	250	Amorphous	86	5.4	4.2	4.2
150	250	Amorphous	90	1.8	0.7	7.9
300	250	Amorphous	94	3.6	-	2.4
25 & RT (#1) [15]	0	Amorphous	93	7.0	-	-
75 & 190 °C (#2) [15]	0	Polycrystalline	93.4	6.6	-	-
0	250 & 190 °C (#3) [41,42]	Polycrystalline	-	-	2.0	98

**Table 6:** FOM values and AR capability of the multicomponent materials developed, as function of the sputtering conditions used. The In_2_O_3_ concentration and the amount of indium-saving are also included.

ITO DCP/AZO RFP	In_2_O_3_ (wt%)/ in-Saving (%)	FOM × 10^−3^ (Ω^−1^)	AR Capability (%)
25/250	67/28	0.35	11.5
75/250	86/7.5	2.23	8.9
150/250	90/3.2	2.22	13.8
300/250	94/-	0.27	11.3
25/-(#1)	93/-	1.03	11.9
75/-(#2)	93.4/-	0.37	12.5
0/250 (#3)	0/100	1.84	13.0

**Table 7:** Compound’s concentrations, estimated by WD-XRF, as function of the used power values. The nature of the film and the (222)/(400) diffraction peak intensity ratio are included.

ITO DC Power (W)	TiO_2_ RF Power (W)	Film Nature	I_(222)_/I_(400)_	In_2_O_3_ (wt%)	TiO_2_ (wt%)	SnO_2_ (wt%)
50	0	Polycrystalline	3.2	92.7	-	7.3
50	25	Polycrystalline	2.9	92.2	0.5	7.3
50	50	Polycrystalline	2.0	91.7	1.1	7.2

**Table 8:** FOM and average T_NIR_ values capability as function of the sputtering conditions used. The In_2_O_3_ concentration, the amount of In-saving and the resistivity of the films are also included.

ITO DCP (W) /TiO_2_ RFP (W)	In_2_O_3_ (wt%)/ in-Saving (%)	FOM × 10^−3^ (Ω^−1^)	T_VIS_ (%)	T_NIR_ (%)	ρ × 10^−4^ (Ω·cm)
0/50	92.7/-	2.45	78.5	43.8	2.38
50/25	92.2/0.5	4.30	85.0	72.9	2.65
50/50	91.7/1.1	8.47	89.0	83.5	1.62

## Data Availability

Not applicable.

## References

[1] Wang Z., Chen C., Wu K., Chong H., Ye H. (2019). Transparent conductive oxides and their applications in near infrared plasmonics. Phys. Stat. Sol..

[2] Rakesh A.A., Nallin S., Maheshwar S., Madhuri S. (2018). Transparent Conducting Oxide Films for Various Applications: A Review. Rev. Adv. Mater. Sci..

[3] Lee N., Bang J.H., Kim H.W., Jeon H. (2021). New approach to SnO_2_-based transparent conducting oxides incorporating synergistic effects of Au nano particles and microwave irradiation. Ceram. Int..

[4] Jayathilake D.S.Y., Nirmal Peiris T.A. (2018). Overview on Transparent Conducting Oxides and State of the Art of Low-cost Doped ZnO Systems. SF J. Mater. Chem. Eng..

[5] Ikhmayies S.J. (2017). Transparent Conducting Oxides for Solar Cell Applications (Chapter 70). Mediterranean Green Buildings & Renewable Energy.

[6] Brunin G., Ricci F., Ha V.A., Rignanese G.M., Hautier G. (2019). Transparent conducting materials discovery using high-throughput computing. NPJ Comput. Mater..

[7] Fortunato E., Ginley D., Hosono H., Paine D.C. (2007). Transparent Conducting Oxides for Photovoltaics. MRS Bull..

[8] Tyagi V.V., Rahim N.A.A., Rahim N.A., Seraj A., Selvaraj L. (2013). Progress in solar PV technology: Research and achievement. Renew. Sust. Ener. Rev..

[9] Luo D., Yang W., Wang Z., Sadhanala A., Hu Q., Su R., Shivanna R., Trindade G.F., Watts J.F., Xu Z. (2018). Enhanced photovoltage for inverted planar heterojunction perovskite solar cells. Science.

[10] Becerril-Romero I., Sylla D., Placidi M., Sánchez Y., Andrade-Arvizu J., Izquierdo-Roca V., Guc M., Pérez-Rodríguez A., Grini S., Vines L. (2020). Transition-Metal Oxides for Kesterite Solar Cells Developed on Transparent Substrates. ACS Appl. Mater. Interfaces.

[11] Txintxurreta J., Berasategui E.G., Ortiz R., Hernández O., Mendizábal L., Barriga J. (2021). Indium Tin Oxide Thin Film Deposition by Magnetron Sputtering at Room Temperature for the Manufacturing of Efficient Transparent Heaters. Coatings.

[12] Winnicki M., Wiatrowski A., Mazur M. (2021). High Power Impulse Magnetron Sputtering of In_2_O_3_/Sn Cold Sprayed Composite Target. Materials.

[13] Ahmeda N.M., Sabah F.A., Abdulgafour H.I., Alsadig A., Suliemane A., Alkhoaryef M. (2019). The effect of post annealing temperature on grain size of indium-tin-oxide for optical and electrical properties improvement. Results Phys..

[14] Sousa M.G., Da Cunha A.F. (2019). Optimization of low temperature RF-magnetron sputtering of indium tin oxide films for solar cell applications. Appl. Surf. Sci..

[15] Fernández S., González J.P., Grandal J., Braña A.F., García F., Borlaf F., Gómez-Mancebo M.B. (2021). Non-treated low temperature indium tin oxide fabricated in oxygen-free environment to low-cost silicon-based solar technology. Vacuum.

[16] Lim J.-W., Na S.-I., Kim H.-K. (2012). Anatase TiO_2_ and ITO co-sputtered films for an indium-saving multicomponent electrode in organic solar cells. Sol. Energy Mater. Sol. Cells.

[17] Fernández S., Borlaf F., García-Pérez F., Gómez-Mancebo M.B., Naranjo F.B., Braña A.F., García-Hernández M., Munuera C. (2019). Tailored amorphous ITAZO transparent conductive electrodes. Mater. Sci. Semicond. Process..

[18] Way A., Luke J., Evans A.D., Li Z., Kim J.-S., Durrant J.R., Hin Lee H.K., Tsoi W.C. (2019). Fluorine doped tin oxide as an alternative of indium tin oxide for bottom electrode of semi-transparent organic photovoltaic devices. AIP Adv..

[19] Yao Z., Duan W., Steuter P., Hüpkes J., Lambertz A., Bittkau K., Pomaska M., Qiu D., Qiu K., Wu Z. (2021). Influence of Oxygen on Sputtered Titanium-Doped Indium Oxide Thin Films and Their Application in Silicon Heterojunction Solar Cells. Sol. RRL.

[20] Jung D.H., Oh Y.-J., Lim S.-H., Kim H.-K., Lee H. (2021). The optical and electrical properties of amorphous gallium/titanium co-doped indium oxide films based on oxygen flow dependence. J. Appl. Phys..

[21] Ciacci L., Werner T.T., Vassura I., Passarini F. (2019). Backlighting the European indium recycling potentials. J. Ind. Ecol..

[22] Palitzsch W. (2018). Implementation of A Circular Economy Based on Recycled, Reused and Recovered Indium, Silicon and Silver Materials for Photovoltaic and Other Applications-Latest News from CABRISS (EU Collaborative Project).

[23] Hutchins M. (2019). The Weekend Read: A Lead-Free Future for Solar PV. PV Mag.

[24] Horcas I., Fernández R., Gomez-Rodriguez J.M., Colchero J., Gómez-Herrero J., Baro A.M. (2007). WSXM: A software for scanning probe microscopy and a tool for nanotechnology. Rev. Sci. Instrum..

[25] Tauc J., Grigorovici R., Vancu A. (1966). Optical properties and electronic structure of Germanium. Phys. Stat. Sol..

[26] Gao M., Job R., De-Sheng X., Fahrner W.R. (2008). Thickness dependence of resistivity and optical reflectance of ITO films. Chin. Phys. Let..

[27] Prepelita P., Filipescu M., Stavarache I., Garoi F., Craciun D. (2017). Transparent thin films of indium tin oxide: Morphology-optical investigations, inter dependence analyses. Appl. Surf. Sci..

[28] Steinecke M., Naran T.A., Christian K., Behrens P., Jensen L., Jupé M., Ristau D. (2021). Electrical and optical properties linked to laser damage behaviour in conductive thin film materials. Opt. Mater. Express.

[29] Zhang K., Zhu F., Huan C.H.A., Wee A.T.S. (2000). Indium tin oxide prepared by radio frequency magnetron sputtering method at a low processing temperature. Thin Solid Films.

[30] Benoy M.D., Mohammed E.M., Suresh Babu M., Binu P.J., Pradeep B. (2009). Thickness dependence of the properties of indium tin oxide (ITO) FILMS prepared by activated reactive evaporation. Braz. J. Phys..

[31] Saeed U., Abdel-Wahab M.S.H., Sajith V.K., Ansari M.S., Ali A.M., Al-Turaif H.A. (2019). Characterization of an amorphous indium tin oxide (ITO) film on a polylactic acid (PLA) substrate. Bull. Mater. Sci..

[32] Mudryi A.V., Ivaniukovich A.V., Ulyashin A.G. (2007). Deposition by magnetron sputtering and characterization of indium tin oxide thin films. Thin Solid Films.

[33] Isiyaku A.K., Ali A.H., Ahmad R.A., Bhari B.Z. (2017). Optoelectronic simulation properties of transparent conducting indium tin oxide for solar cell application. J. Sci. Tech..

[34] Oh W.-K., Hussain S.Q., Lee Y.-J., Lee Y., Ahn S., Yi J. (2012). Study on the ITO work function and hole injection barrier at the interface of ITO/a-Si:H(p) in amorphous/crystalline silicon heterojunction solar cells. Mater. Res. Bull..

[35] Klein A., Körber C., Wachau A., Säuberlich F., Gassenbauer Y., Harvey S.P., Proffit D.E., Mason T.O. (2010). Transparent conducting oxides for photovoltaics: Manipulation of fermi level, work function and energy band alignment. Materials.

[36] Wu C.-C., Diao C.-C. Effects of substrate temperature on the properties of the indium tin oxide thin films deposited by sputtering method. Proceedings of the 13th MATEC Web of Conferences.

[37] Louwen A., Van Sark W., Schropp R., Faaij A. (2016). A cost roadmap for silicon heterojunction solar cells. Sol. Energy Mater. Sol. Cells.

[38] Minemoto T., Mizuta T., Takakura H., Hamakawa Y. (2007). Antireflective coating fabricated by chemical deposition of ZnO for spherical Si solar cells. Sol. Energy Mater. Sol. Cells.

[39] Le A.H.T., Dao V.A., Pham D.P., Kim S., Dutta S., Thi Nguyen C.P., Lee Y., Kim Y., Yi J. (2019). Damage to passivation contact in silicon heterojunction solar cells by ITO sputtering under various plasma excitation modes. Sol. Energy Mater. Sol. Cells.

[40] Meza D., Cruz A., Morales-Vilches A.B., Korte L., Stannowski B. (2019). Aluminum-Doped Zinc Oxide as Front Electrode for Rear Emitter Silicon Heterojunction Solar Cells with High Efficiency. Appl. Sci..

[41] Fernández S., Bosca A., Pedros J., Ines A., Fernández M., Arnedo I., González J.P., de la Cruz M., Sanz D., Molinero A. (2019). Advanced Graphene-Based Transparent Conductive Electrodes for Photovoltaic Applications. Micromachines.

[42] Fernández S., García-Pérez Borlaf F., Gómez-Mancebo M.B., Braña A.F., Naranjo F.B., García-Hernández M., Munuera C. (2018). Amorphous ITAZO films as advanced coatings for cost-effective silicon based photovoltaic device technology. Mater. Today Proc..

[43] Taylor M.P., Readey D.W., Van Hest M.F.A.M., Teplin C.W., Alleman J.L., Dabney M.S., Gedvilas L.M., Keyes B.M., To B., Perkins J.D. (2008). The Remarkable Thermal Stability of Amorphous In-Zn-O Transparent Conductors. Adv. Funct. Mater..

[44] Hashimoto R., Abe Y., Nakada T. (2008). High Mobility Titanium-Doped In_2_O_3_ Thin Films Prepared by Sputtering/Post-Annealing Technique. Appl. Phys. Express.

[45] Bowers J.W., Upadhyaya H.M., Nakada T., Tiwari A.N. (2010). Effects of surface treatments on high mobility ITiO coated glass substrates for dye sensitized solar cells and their tandem solar cell applications. Sol. Energy Mater. Sol. Cell.

